# Changes in DNA methylation associated with a specific mode of delivery: a pilot study

**DOI:** 10.3389/fmed.2024.1291429

**Published:** 2024-01-18

**Authors:** Patrik Krumpolec, Dominik Kodada, Dominik Hadžega, Oliver Petrovič, Klaudia Babišová, Erik Dosedla, Zuzana Turcsányiová, Gabriel Minárik

**Affiliations:** ^1^Medirex Group Academy n.o., Nitra, Slovakia; ^2^Department of Clinical Biology, Genetics and Clinical Genetics, Faculty of Medicine, Comenius University in Bratislava, Bratislava, Slovakia; ^3^Department of Gynaecology and Obstetrics, Faculty of Medicine, Pavol Jozef Šafárik University in Košice, Košice, Slovakia

**Keywords:** DNA methylation, 5-methyl cytosine, CpG sites, vaginal delivery, caesarean section

## Abstract

**Background:**

The mode of delivery represents an epigenetic factor with potential to affect further development of the individual by multiple mechanisms. DNA methylation may be one of them, representing a major epigenetic mechanism involving direct chemical modification of the individual’s DNA. This pilot study aims to examine whether a specific mode of delivery induces changes of DNA methylation by comparing the umbilical cord blood and peripheral blood of the newborns.

**Methods:**

Blood samples from infants born by vaginal delivery and caesarean section were analysed to prepare the Methylseq library according to NEBNext enzymatic Methyl-seq Methylation Library Preparation Kit with further generation of target-enriched DNA libraries using the Twist Human Methylome Panel. DNA methylation status was determined using Illumina next-generation sequencing (NGS).

**Results:**

We identified 168 differentially methylated regions in umbilical cord blood samples and 157 regions in peripheral blood samples. These were associated with 59 common biological, metabolic and signalling pathways for umbilical cord and peripheral blood samples.

**Conclusion:**

Caesarean section is likely to represent an important epigenetic factor with the potential to induce changes in the genome that could play an important role in development of a broad spectrum of disorders. Our results could contribute to the elucidation of how epigenetic factors, such as a specific mode of delivery, could have adverse impact on health of an individual later in their life.

## Introduction

In the most recent three decades, a growing number of deliveries by caesarean section (CS) has been observed as the worldwide rate has risen from 7% in 1990 to about 21% at present (1, 2). In countries such as the Dominican Republic, Brazil, Cyprus, Egypt and Turkey, CSs even outnumber vaginal deliveries (VDs). Moreover, this number continues to increase as about 29% of all births are likely to be performed using caesarean section by 2030 (2).

Along with the rising rate of CSs, there has also been a trend of an increased share of CS due to non-clinical reasons (maternal preference or other social factors). The excessive use of CS has raised public health concerns (3) as an increasing volume of evidence indicates long-term impact of CS on the maternal and also the child’s state of health. The molecular mechanisms involved in outcomes of CS are not entirely explained (4, 5), but an important role could play an infant stress during labour. Although it represents an advantage in preparation for extrauterine life, the stress experienced by infants varies vastly between delivery modes (6). There is a flush of stress hormones in the infant during VD. Especially hormones like catecholamines and cortisol are responsible for readiness of the respiratory system for gas exchange, increased blood flow, activation of the CNS and mobilization of fuel processing (7, 8). However, this high expression of stress hormones also triggers a cascade of hormones and cytokines involved in inflammatory defence pathways (7, 9). Compared to CS, infants born via VD have higher levels of such hormone release (7, 8, 10, 11), greater complexity of intestinal flora (12) and higher immune response (13). It is therefore not surprising that epidemiological studies showed that children delivered by CS are more likely to develop respiratory disorders, neurological diseases (e.g., autism spectrum disorders, schizophrenia) (14, 15) and immune-related diseases (e.g., asthma, skin atopy, coeliac disease and juvenile arthritis, type 1 diabetes or obesity) (1, 16–22).

Although the involved biological mechanisms are not yet completely understood, recent evidence suggests that epigenetic alterations (e.g., changes in DNA methylation, histone modifications and non-coding RNA) play a certain role.

DNA methylation is an epigenetic process in which non-sequence-based regulatory information can be mitotically transferred between the mother and daughter cells. In this process, a methyl group is added at the 5-position of a cytosine-guanine (CpG) dinucleotide. Methylated cytosines can be distributed within the genome on CpG islands, shores, shelves and in the so-called open sea regions (23). Shores are regions within 2 kb from CpG islands, shelves are regions 2–4 kb from CpG islands and open sea regions are isolated CpG sites in the genome without a specific designation or without any kind of CpG content enrichment (referred in the literature also as other regions) (24).

DNA methylation can be associated with inhibition of gene expression by interference with transcription binding proteins, which influences chromatin remodelling (25). About 90% of all methylated CpG sites are localized in transposable, repetitive elements, while others occur in gene promoters. During early mammalian embryogenesis, DNA methylation patterns are completely reset with further *de novo* methylation and demethylation events allowing tissue differentiation (26). Almost all CpG sites are *de novo* methylated except for the sites labelled as CpG islands that are protected by virtue of cis-acting regulatory sequences (27). To a certain extent, all further changes in DNA methylation are conserved and gene-specific. For example, pluripotency genes become *de novo* methylated at the time of gastrulation, while tissue-specific regulatory regions undergo demethylation during embryonic development or during adult stem cell differentiation (28–31). It was believed that DNA methylation patterns created during development do not undergo further modifications, however, recent studies showed that methylation could occur also postnatally in terminally differentiated tissues. This can be induced by internal signalization [e.g., DNA methylation changes in the liver induced by testosterone (32–34)] as well as by effect of environment or due to specific behaviors which represent epigenetic factors (35). Moreover, after onset, such changes can persist for extended time periods (27).

It appears that the mode of delivery represents one of the crucial epigenetic events associated with changes in DNA methylation that could have profound impact on human health in later stages of life.

This study provides evaluation how the mode of delivery may induce changes in newborn’s genome that can potentially lead to a broad spectrum of health-related complications in the future. We have also identified biological and metabolic pathways that could be affected by differences in DNA methylation as the major driver of such delivery-dependent modifications in the genome.

To our knowledge, there are only a few works that analyze dynamics of changes in DNA methylation in early postnatal life. Our results indicate that changes in DNA methylation (i) occur not only at the time of birth, as was described in the literature so far, (ii) but also postnatally and (ii) there could be mechanism to adopt to such changes. Therefore our work has pointed out the need of continual study of changes in DNA methylation during the life to understand their mechanisms and potential consequences.

## Methods

### Demographic and clinical data

The study includes 12 healthy newborn infants from the Slovakian population. All of them were delivered in full term as singletons. Exclusion criteria were multiple pregnancies, preterm delivery (gestational age <37 weeks), pregnancy resulting from *in vitro* fertilisation, maternal diabetes, preeclampsia, congenital infection and chromosomal disorders. We compared two modes of delivery: vaginal delivery (VD; *n* = 7) and elective caesarean section (CS; *n* = 5). In two cases, vaginal delivery was induced.

### Samples

From each study subject, 5 mL EDTA blood from the umbilical cord was sampled upon delivery and 2 mL EDTA blood from a peripheral vein on 2nd/3rd day of infants’ postnatal age. Sample analyses were performed separately as on the two sub-groups comprising umbilical cord and peripheral blood (as two separate single time-point analyses). In the next step, a comparison was performed on these two different datasets based on delivery mode (CS vs. VD). We evaluated also changes in DNA methylation in time, i.e., upon delivery and on 2nd/3rd day after delivery.

### DNA preparation

Genomic DNA was extracted using DNeasy Blood & Tissue Kit (QIAGEN) following the manufacturer’s instructions. DNA quantification was performed with Qubit dsDNA Broad Range Quantitation Assay (Thermo Fisher Scientific).

Genomic DNA (with the volume of 100 μL) was sheared using a focused ultrasound on Covaris S220 system (sonication conditions were optimized, the applied settings are shown in the Table 1). Ultrasonication-based DNA fragmentation was selected to avoid the removal of methyl groups from DNA by enzymatic fragmentation. Fragmented genomic DNA was cleaned up using 1.2× sample volume of AMPure XP beads (Beckman Coulter, United States).

**Table 1:** Summary of Covaris operating conditions.

Parameter	Value/mode
Duty factor	10%
Peak incidence power	175 Watt
Cycles per burst	200
Time	280 ms
Water bath temperature	10°C
Water level	12
Power mode	Frequency sweeping
Degassing mode	Continuous

### MethylSeq library preparations

Only the samples with suitable size (average of 240–290 bp) were allowed to proceed to preparation of the MethylSeq library pursuant to NEBNext enzymatic Methyl-seq Methylation Library Preparation Kit (Twist Bioscience). These were used to generate target-enriched DNA libraries by Twist Human Methylome Panel (Twist Bioscience).

Mechanically sheared DNA underwent end repair. After ligation of methylated adapters, 5-methylcytosines were oxidized by TET2 enzyme and in further APOBEC treatment, unmethylated cytosines were deaminated to uraciles. The converted library was then indexed by PCR amplification. After quality check, eight libraries were pooled in equimolar quantity with further overnight hybridization with a custom double-stranded DNA panel to target specific regions. Fragments of interest were captured with streptavidin binding beads. The pool of libraries was amplified by qPCR. Quantification and validation of enriched libraries was performed by Qubit dsDNA High Sensitivity Quantitation Assay (Thermo Fisher Scientific) and Agilent Bioanalyzer High Sensitivity DNA kit. Out of the 16 original pairs of samples, 12 pairs qualified for sequencing on Illumina next-generation sequencing (NGS) systems (NextSeq 500 and NextSeq 2000 by Illumina) using 2 × 75 or 2 × 100 paired-end sequencing kits and protocols, respectively.

### MethylSeq data analysis

The entire analysis was performed using the BaseSpace Sequence Hub (BSSH) cloud service. In the first step, the BCL files were converted into FASTQ files. Next, FASTQ files were submitted as the input to the DRAGEN Methylation pipeline. A conversion of the bases from C-to-T, and G-to-A was then performed. The bisulfite converted reads were afterwards aligned to the reference genome, in our case it was the hg19 alt-aware UCSC reference genome already present in the BSSH. Targeted methylation calling was performed once the converted reads were aligned to the reference genome. The regions subjected to the analysis were provided in the BED file from Twist Bioscience. In the background, BSSH utilizes the Bismark Bisulfite Read Mapper to map the reads and to identify the local cytosine methylation status. As a result, we obtained the BAM files as well as the Cytosine Report TXT file, which then served as an input to the MethylKit application.

The MethylKit application within the BSSH represents the second phase of the computational analysis. Here, the input data representing the output from the previous phase were provided. At the same time, the conceptual division was determined between the samples considered experimental and the samples used as a reference set.

### Methylation pathway analysis

Pathway analysis was based on genes where differentially methylated CpG sites were observed. The list of these genes was compared against the Kyoto Encyclopaedia of Genes and Genomes (KEGG) with the aim to identify biological, metabolic and signalling pathways that could be related to genes affected by changes in methylation.

### Data analysis

Statistical analyses were performed using SAS Jump Statistics Software (United States) and MS Excel software. Data are presented as mean values ± SD. Paired *t*-test was used to assess the difference between the VD group and the CS group. Pearson correlation was used to determine the association state between variables. Statistical significance was considered at *p* < 0.05, and for trends, *p* < 0.10.

## Results

### Descriptive statistics

The study includes 12 healthy newborn infants delivered in full term as singletons. Detailed characteristics of the study groups are available in the Table 2. Seven newborns were delivered by VD, while five newborns by elective CS. In two cases, the vaginal delivery was induced. None of the CS represented an emergency. Maternal age ranged from 24 to 37 years, none of mothers have a history of smoking. Ten mothers were of Slovak ethnic origin, one was of Albanian and one of Polish ethnic origin. Two cases in the VD group and one infant in the CS group recquired artificial feeding without any other complications. In one case in the VD group, partial syndaktyl was diagnosed.

**Table 2:** Characteristics of the study groups.

	Entire study population	Vaginal delivery	Caesarean section	*p*-value
*N*	12	7	5	—
Age (mother) (year)	31.8 ± 3.8	31.4 ± 3.7	32 ± 4.1	0.800
BMI (mother) (kg.m^−2^)	27.1 ± 2.9	26.6 ± 2.1	27.8 ± 4.0	0.497
GA week at delivery	39.5 ± 1.2	39.7 ± 0.8	39.2 ± 1.6	0.479
Delivery weight (g)	3281.7 ± 345.7	3134.3 ± 170.5	3488 ± 440.3	0.078^†^
Sex (F/M)	10/2	6/1	4/1	—

Data are expressed as mean value ± SD; *p*-value indicates difference between vaginal delivery (VD) group and caesarean section CS group; ^†^denotes trend *p* < 0.1 (36).

We did not observe any significant differences between the VD group and the CS group, except for higher delivery weight in the CS group, which has a character of a trend (*p* = 0.078). The magnitude of correlation between observed parameters was low (data not shown).

### Methylation distribution in umbilical cord blood samples

**Figure 1:**
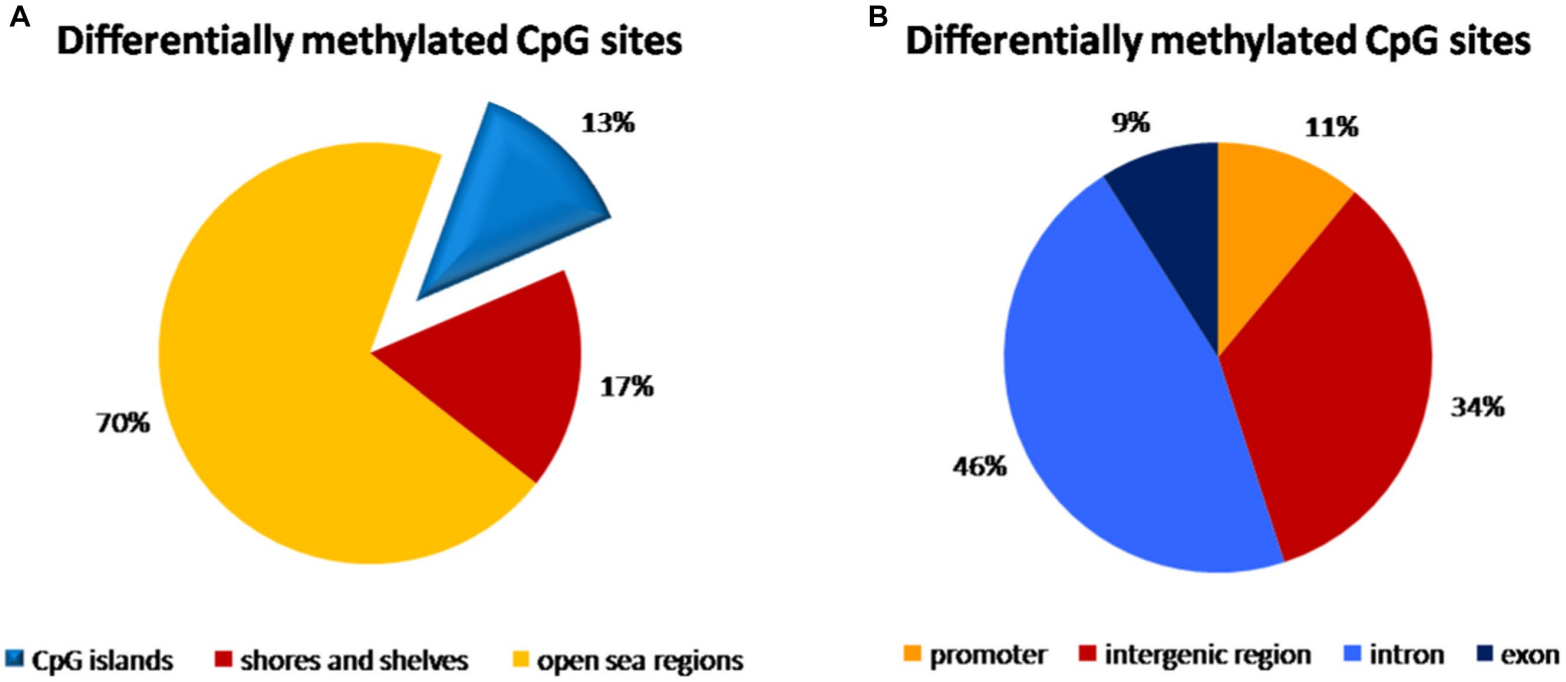
Distribution of differentially methylated CpG dinucleotide in the umbilical cord blood samples according to neighbourhood context **(A)** and functional genomic distribution **(B)**. **(A)** A predominant portion of differentially methylated CpG probe sites (70%) was observed in open sea regions, 13% on the CpG islands and 17% on the shores and shelves. **(B)** The highest distribution of differentially methylated CpG probe sites was found within introns (46%) and in the intergenic regions (34%), while only 11% of CpG probe sites with different methylation were localized within promoters and 9% in exons.

In the umbilical cord blood samples, the total of 3,595,242 CpG sites was compared for all analyzed samples. In the comparison of vaginal delivery and caesarean section, from the total number of 4,352 differentially methylated bases, 1,776 bases were hypermethylated, while 2,576 bases were hypomethylated. Three thousand seventeen bases (69.3%) were assigned as significantly differentially methylated. These were localized into 168 differentially methylated regions, whereas 78 regions were hypermethylated and 90 regions were hypomethylated. As shown in the Figure 1A, 13% of methylation CpG probe sites differences were found on the CpG islands, 17% on the shores and shelves and a predominant portion of CpG sites was in open sea regions. The highest distribution of differentially methylated CpG sites was observed within introns (46%) and in the intergenic regions (34%), while only 11 and 9% of CpG sites with different methylation were localized within promoters and exons, respectively (Figure 1B).

### Methylation distribution in peripheral blood samples

**Figure 2:**
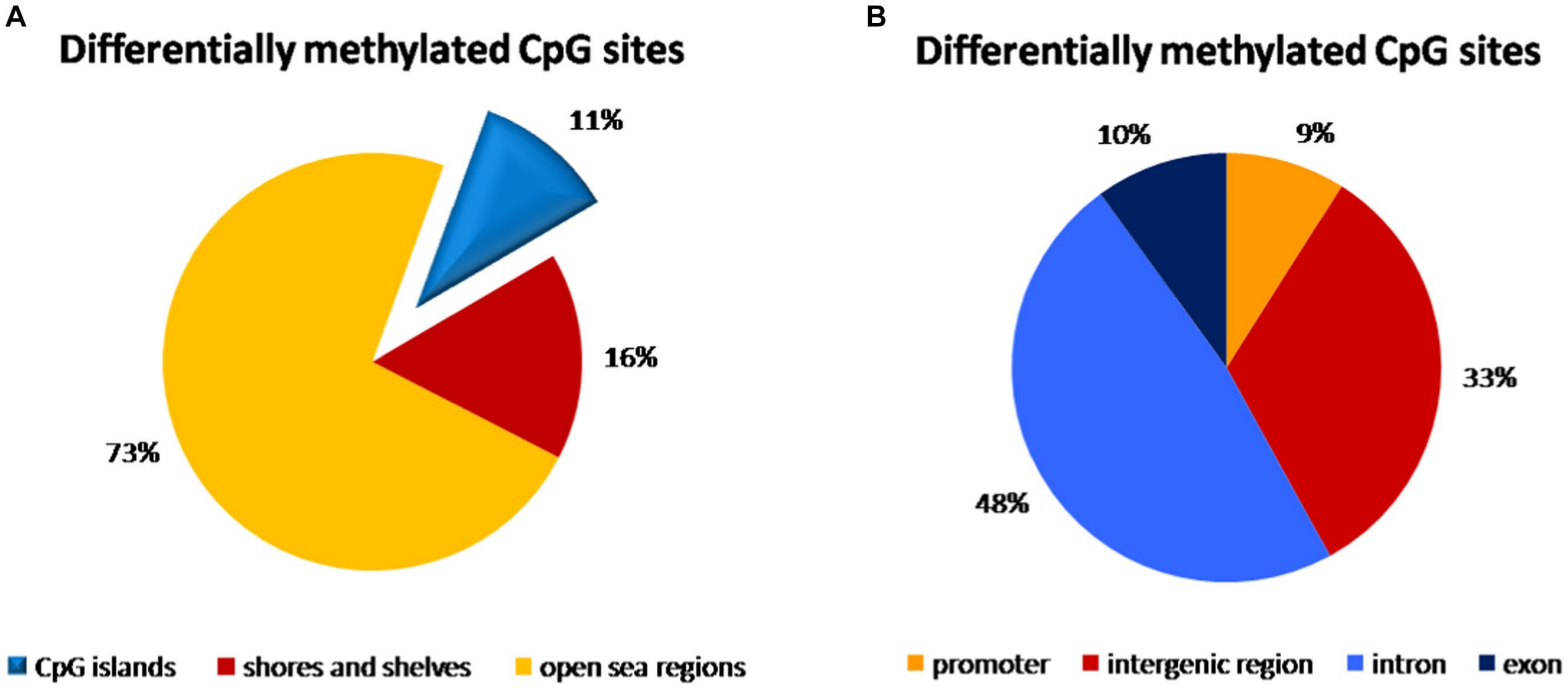
Distribution of differentially methylated CpG dinucleotide in the peripheral blood samples according to neighbourhood context **(A)** and functional genomic distribution **(B)**. **(A)** Almost three quarters (73%) of all CpG probe sites with different methylation were identified in open sea regions of the genome, 11% were localized on CpG islands and 16% were found on shores and shelves. **(B)** The highest distribution of differentially methylated CpG probe sites was found within introns (48%) and in the intergenic regions (33%), while only 10% of CpG probe sites with different methylation were localized in exons and only 9% in promoters.

We compared 3,227,047 CpG sites common for all samples of peripheral blood. In the comparison of vaginal delivery and caesarean section, 3,853 bases were methylated differentially. Specifically, 1,873 bases were hypermethylated while 1,980 were hypomethylated. However, only 55.1% of them were assigned as significantly differentially methylated. We identified 79 hypermethylated and 78 hypomethylated regions, i.e., 157 regions showed differential methylation. Almost three quarters (73%) of all CpG sites with different methylation were identified in open sea regions of the genome, whereas 11% were localized on CpG islands and 16% were found on shores and shelves (Figure 2A). Introns accounted for 48% of differentially methylated CpG sites, 33% was in intergenic regions, while 10% in exons and only 9% in promoters (Figure 2B).

### Chromosomal location of differentially methylated CpG sites

**Figure 3:**
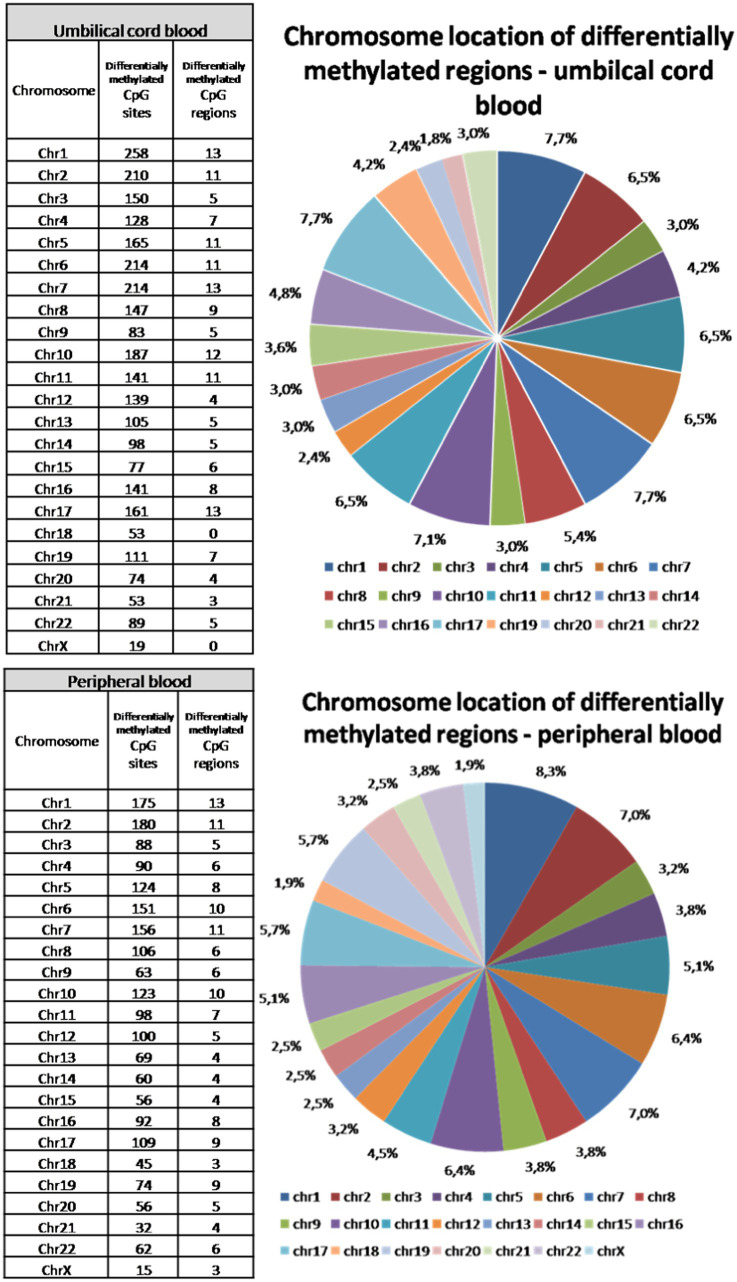
Chromosomal location of differentially methylated regions in the umbilical cord and peripheral blood samples. Differences in methylation was observed in all somatic chromosomes and also on X chromosome in peripheral blood samples, while there were no differences in methylation of chromosome 18 and chromosome X in umbilical cord blood samples. The highest proportion of different methylation was identified on chromosomes 1, 2, 6, and 7. The lowest rate of differentially methylated CpG sites was on chromosomes 18 and 21 and on X chromosome.

Although peripheral blood samples were obtained several days after delivery, the total number of all differentially methylated CpG sites is only about 10% lower than in umbilical cord blood samples. Final deviation between significantly differently methylated CpG sites across both types of samples reached more than 30%. Different methylation was approved in all somatic chromosomes and on X chromosome in peripheral blood samples. In umbilical cord blood samples, no significant changes in methylation of chromosome 18 and chromosome X were observed between vaginal delivery and caesarean section samples (Figure 3). The highest proportion of different methylation was identified on chromosomes 1, 2, 6 and 7 (about 7–8.5%). The lowest rate of differentially methylated CpG sites was on chromosomes 18 and 21 and on X chromosome (up to 1.8%).

### Methylation and pathway analysis

**Figure 4:**
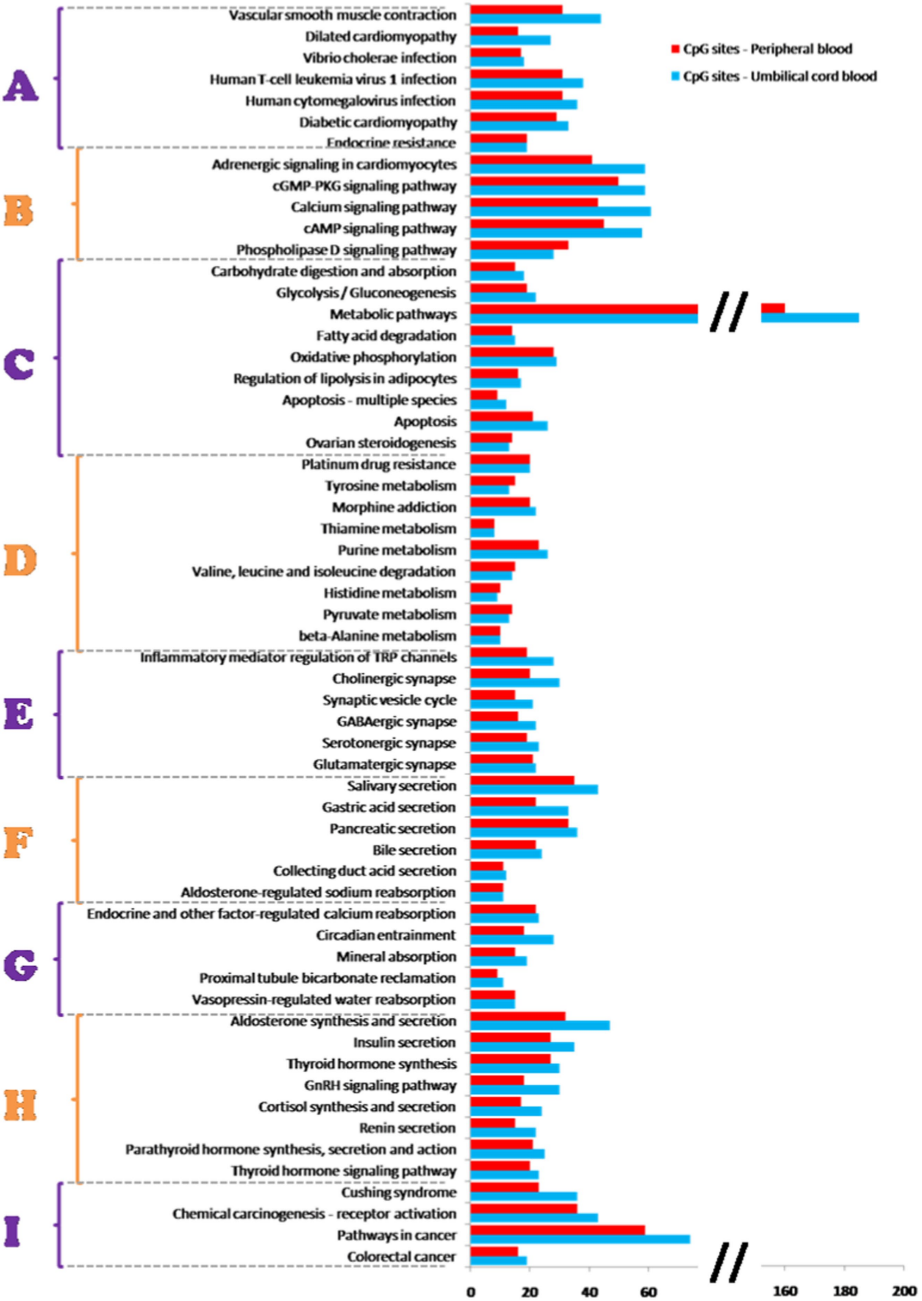
Methylation pathway analysis. Fifty-nine common biological, metabolic and signalling pathways for umbilical cord blood samples and peripheral blood samples identified based on a comparison of KEGG database with the analyzed genes affected by mode of delivery-related differences in DNA methylation. Pathways were grouped into 9 categories **(A–I)**: **A**—diseases, **B**—signalling pathways, **C**—metabolic pathways, **D**—pathways of specific metabolites, **E**—synaptic pathways, **F**—synthesis and secretion of body fluids, **G**—mineral management, **H**—hormonal metabolism, **I**—carcinogenesis. Pathways are ordered with respect to their significance (from higher to lower). Significance based on g:SCS-adjusted *p*-value <0.05 (37).

Based on genes affected by different methylation, we identified 59 common biological, metabolic and signalling pathways for umbilical cord and peripheral blood samples (Figure 4). In addition to the above, 20 pathways were assigned only to umbilical cord blood samples and 10 pathways only to peripheral blood samples (Table 3).

**Table 3:** Methylation pathway analysis.

KEGG Biological pathway	CpG sites with different methylation	Affected genes
*Umbilical cord blood*		
Nitrogen metabolism	11	CPS1
Alanine, aspartate and glutamate metabolism	11	CPS1, ADSS1, ABAT
Endometrial cancer	15	EGF, CTNNA1, MYC, CTNNA2, POLK, TCF7L1
GnRH secretion	16	PRKCB, PRKCA, CACNA1I, ITPR3, CACNA1H, GNA11, ITPR1
Amphetamine addiction	18	PRKCB, PRKCA, GNAS, CREB5, SLC18A2
Arrhythmogenic right ventricular cardiomyopathy	18	CTNNA1, SGCA, CACNG7, CTNNA2, TCF7L1, LAMA2
Long-term potentiation	19	PRKCB, PRKCA, ITPR3, ITPR1
TGF-beta signalling pathway	19	MYC, THSD4, TFDP1, SMAD3, TGIF1, ACVR2A, TGIF2
Glioma	21	PRKCB, EGF, PRKCA, PLCG2, POLK
HIF-1 signalling pathway	21	PRKCB, EGF, PRKCA, PLCG2, VEGFA, HK2, HK1, PFKP
Cardiac muscle contraction	23	CACNG7, TPM4
Melanogenesis	23	PRKCB, PRKCA, WNT8A, GNAS, TCF7L1, FZD1
Complement and coagulation cascades	25	SERPINA5
Neurotrophin signalling pathway	25	PLCG2, NFKBIB, TP73
Growth hormone synthesis, secretion and action	27	GHRHR, SSTR5, ITPR3, GNAS, CREB5, GNA11, ITPR1
Dopaminergic synapse	27	PRKCB, PRKCA, ITPR3, GNAS, CREB5, GNAL, SLC18A2, ITPR1
Hippo signalling pathway	27	CTNNA1, MYC, SMAD3, CTNNA2, WNT8A, TCF7L1, FZD1, WWC1, GLI2, TP73
Alcoholic liver disease	33	ACADVL, ACACB, LPIN1, ACADM, TCF7L1, IL17RC, IL17RA
Oxytocin signalling pathway	37	PRKCB, PRKCA, TRPM2, CACNG7, ITPR3, GNAS, PLA2G4B, JMJD7-PLA2G4B, ITPR1
Prion disease	41	KLC3, ITPR3, HSPA6, CREB5, SDHA, NDUFA9, PSMD1, ITPR1
*Peripheral blood*		
Tryptophan metabolism	11	MAOA, IDO2, INMT, HADH
Hedgehog signalling pathway	12	IQCE, BOC, EVC, DISP1, MGRN1, GLI2
Fatty acid degradation	14	HADHB, HADH
Drug metabolism—cytochrome P450	14	MAOA, GSTM5, MGST1
Fc gamma R-mediated phagocytosis	17	PLD2, SYK, DOCK1, PLA2G4B, JMJD7-PLA2G4B, PRKCA, ASAP2, SCIN, PRKCE, INPP5D, PRKCB, SPHK1
AGE-RAGE signalling pathway in diabetic complications	17	PLCE1, NFATC1, PRKCA, SMAD3, PRKCE, TGFBR2, PRKCB, COL4A2, NOS3
Platelet activation	19	TBXAS1, SYK, ITPR3, PLA2G4B, JMJD7-PLA2G4B, GNAS, NOS3, ARHGEF12
Relaxin signalling pathway	20	GNAS, PRKCA, CREB5, GNG10, TGFBR2, COL4A2, NOS3
Apelin signalling pathway	21	ITPR3, SMAD3, PRKCE, GNG10, NOS3, MAP1LC3B2, SPHK1
Endocytosis	36	PLD2, CXCR2, HLA-G, NEDD4L, AGAP1, EPN3, IGF1R, IQSEC3, STAM, GRK5, PDGFRA, PSD4, HLA-A, CHMP4B, SMAD3, MVB12B, RAB31, ASAP2, TGFBR2, DNM3, RAB5A

Biological, metabolic and signalling pathways for umbilical cord blood samples and peripheral blood samples identified based on a comparison of KEGG database with the analyzed genes affected by mode of delivery-related differences in DNA methylation.

## Discussion

In our analyses comparing DNA methylation between samples from newborns delivered via conventional vaginal delivery and via caesarean section, we identified 168 regions in umbilical cord blood samples as well as 157 regions in peripheral blood samples with differential methylation. This indicates that mode of delivery could have profound impact on a wide range of genes and that caesarean section may represent an epigenetic factor with impact on DNA methylation state. Moreover, we also identified 59 biological pathways that are associated with genes from differentially methylated regions and not only at the time of birth but also later after the delivery. Such observation confirmed the ability of an epigenetic factor to change methylation status established during *in utero* development. Our observation that a considerable number of biological and signalling pathways are still changed even several days after delivery confirms the potential of persistence of induced changes. This process then creates space and time to induce further changes that could have adverse affect on the individual’s health status later in the life. Further research is needed to elucidate whether or not such changes persist for a longer time after delivery, or they are reversible.

Hypothesis of methylation state plasticity after delivery could be confirmed also by normalization of differences in methylation status in case of 21 biological pathways that disappeared after several days. On the other hand, we simultaneously identified other changes in DNA methylation state that could be associated with the need to amend the adverse state in newborns delivered by caesarean section (Table 3).

Identified pathways can be stratified into 9 categories based on affected processes and/or functions, as is shown in the Figure 4. Whereas almost none of the items in this categorization do not work as single separate units but are mutually interconnected, we try to discuss their role as components of several disorders and diseases which seem to be affected by changes in DNA methylation.

As shown in Figure 4, one of the pathways most affected by differential methylation is the Human cytomegalovirus (HCMV) infection pathway. HCMV infection is one of the most common infectious cause of congenital defects (38). About one fifth of children with congenital HCMV suffer from HCMV symptoms or long-term health problems, such as motor and intellectual disability, liver, lung and growth complication while hearing loss is the most common complication after congenital HCMV infection (39). In particularly severe cases, an HCMV infection can lead to pregnancy loss. It was observed that HCMV infection resulted in the re-localization of DNMT1 and DNMT3B from the nucleus in the cytoplasm. Such a draining of DNA methyltransferases from the nucleus may provide the opportunity to express viral proteins and replicate viral genome in a DNA methylation-free environment without formation of supressing complexes on viral promoters rather than by direct interaction with DNMTs (40). There could also be the possibility that HCMV could manipulate natural processes of the host cell designed to keep DNMTs outside of the nucleus because active retention of DNMT1 in the cytoplasm was also observed during early embryogenesis (41). Thus, our observation of potential relation between the changes in DNA methylation machinery and HCMV pathways suggests a potentially higher risk of developing symptoms or complications associated with HCMV infection in children delivered by caesarean section.

DNA methylation is considered as a host defence mechanism for inactivation of retrovirus expression (42). The first discovered human retrovirus was Human T-cell leukaemia virus type 1 (HTLV-1), which is associated with T-cell lymphoproliferative disorders (e.g., adult T-cell leukaemia/lymphoma, ATLL) and inflammatory diseases (e.g., HTLV-1-associated myelopathy, pneumonitis, uveitis, arthritis and myositis) (43). ATLL genomes are characterized by a prominent promoter-related CpG islands and DNA hypermethylation. Moreover, the level of hypermethylation is correlated with poor prognosis (44). Mutations in epigenetic regulators of DNA methylation TET methylcytosine dioxygenase 2 (TET2) and mixed-lineage leukaemia protein 3 (MLL3) were observed in ATLL patients, as well as CpG hypermethylation in gene encoding zinc finger transcription factors and major histocompatibility class I proteins (44, 45).

Nowadays, it is still unclear why ATLL takes decades to manifest itself, why only 5–10% of infected subjects develop symptoms and how HTLV-1-associated diseases can be prevented and treated. It is suggested that most individuals who develop ATLL in adult age were infected already as infants, probably via breast feeding (46). In the light of our observation of differences in DNA methylation between newborns delivered via VD and CS, we suggest that CS-related differences in DNA methylation could lead to higher susceptibility to development of ATLL symptoms and manifestations and that there is higher probability of HTLV-1 expression reactivation. Such a hypothesis requires further study whether or not CS could be associated with higher incidence of ATLL manifestation.

To date, multiple publications supported the importance of DNA methylation in a broad spectrum of cardiovascular diseases, including atherogenesis (47), coronary artery diseases (48, 49), dilated cardiomyopathy (50) and heart failure (51–53). In our analysis, we identified three pathways that could be potentially associated with different methylation (Figure 4). Vascular smooth muscle cells (VSMCs) are involved in maintaining the structural integrity and physiological function of blood vessels, regulating blood pressure and controlling vascular contraction and relaxation (54, 55). As individuals age, VSMCs suffer from mechanical stimulation, chronic inflammation, calcifications and other epigenetic events (56). The VSMCs proliferation is one of the primary features of vascular ageing-related diseases. Cell proliferation is tightly controlled by DNA methylation via regulation of gene transcription. DNMTs inhibit gene expression by promoting DNA methylation and regulating the proliferation of VSMCs. It has been observed that as DNA demethylation of PDGF increases PDGR mRNA and protein expression, while the hypomethylation of HIF-1α causes the proliferation and migration of VSMCs (57, 58). On the contrary, DNA hypermethylation of MFN2, PTEN and ER-α causes low expression of MFN2, PTEN and ER-α that leads also to the proliferation of VSMCs (59–61). Liu et al. (62) demonstrated that vascular injury and vascular disorders are associated with downregulation of expression of TET2, an enzyme with a key role in the DNA demethylation pathway. Abnormal DNA methylation and DNMTs suppression are also tightly linked to vascular diseases (63). DNA hypermethylation regulates cardio-metabolism by destroying nuclear respiratory factor 1-dependent oxidative metabolism (64). Moreover, in heart failure patients upregulation of DNMT3a and DNMT3b expression was observed (64). Additionally, in patients with dilated cardiomyopathies, association of aberrant DNA methylation was observed with significant differences in expression of ADORA2A and LY75 mRNA (50). Summarising the above, changes in DNA methylation potentially resulting from delivery by caesarean section associated with adverse changes in genome could increase the risk of developing cardiovascular complications later in the life.

Defects in biosynthesis and secretion of aldosterone, which represent another pathway affected by differentially methylated CpG islands in our analysis, can lead to sodium processing disorders manifested as hypotension, hyponatraemia, hyperkalaemia and acidosis. Aldosterone is important for regulation of sodium conservation in the kidney, colon, salivary and sweat glands. This adrenal steroid hormone acts via the mineralocorticoid receptor to promote active transport of sodium and excretion of potassium through activation of amiloride-sensitive sodium channels and a Na/K-ATP-ase pump (65). For example, patients with Addison’s disease are hyponatraemic and hyperkaliaemic because of potassium retention and excessive urine excretion of sodium (66). Thus, our observation of the association of different methylation with aldosterone-regulated sodium reabsorption is in compliance with reported sodium transport disorders. Gene expression of aldosterone synthase CYP11B2 is epigenetically controlled (67). Takeda et al. (68) show that infusion of angiotensin II in rats decreased the methylation ratio of the CYP11B2 gene and increased gene expression in the adrenal gland. They also observed that a low-sodium diet induced hypomethylation of CYP11B2 and increased its mRNA level parallel with synthesis of aldosterone. Moreover, aldosterone biosynthesis also includes the cAMP signalling pathway which is one of the pathways most affected by DNA methylation in our analysis (Figure 4).

As mentioned above, caesarean section is a potential epigenetic stress factor. In their work, Zhang et al. (69) tested hypothesis that chronic negative stress leads to alterations in DNA methylation of certain cardiac genes with further pathologic remodelling of the heart. The authors approved that altered methylation on specific genes is associated with the adrenergic signalling of cardiomyocyte pathways (e.g., via adrenergic receptor-α1) resulted in induced cardiac remodelling and arrhythmias. Also Xiao et al. (70) reported that abnormal DNA methylation within the CpG islands of the promoter region of voltage-gated potassium channel subfamily E genes has been linked to cardiac arrhythmias. We also found association of differentially methylated CpG sites with the pathway of adrenergic signalling in cardiomyocytes as well as with dilated cardiomyopathy pathway. Thus, changes in DNA methylation related to caesarean section can be tightly associated with cardiac dysfunction.

Another signalling pathway that could be influenced by modulation of DNA methylation state is cAMP signalling pathway. Adenosine 3′,5′-cyclic monophosphate (cAMP) is a highly regulated secondary messenger involved in numerous biological processes (71). Although it is known that chronic activation of the cAMP pathway results in cardiac hypertrophy and fibrosis, the underlying mechanisms linking elevated cAMP levels to cardiomyopathy are not fully elucidated. Fang et al. demonstrated that elevated level of cAMP analog *N*^6^,2′-*O*-dibutyryladenosine 3′,5′-cyclic monophosphate (DBcAMP) changed the expression of DNMTs and TETs as enzymes regulating genomic DNA methylation levels. Such a change induced increased global DNA methylation in HL-1 cardiomyocytes. Thus, DNA methylation mediates the upregulation of hypertrophy cardiomyopathy genes induced by cAMP. Possible explanation could involve physiological activation of the cAMP-dependent protein kinase (PKA) leading to phosphorylation of numerous transcription factors, including the cAMP response element-binding protein (CREB) (72). CREB then binds to cAMP response elements (CREs) on gene promoters and activates expression of involved genes. Similarly, during chronic activation of the cAMP pathway, increased cAMP induced hypermethylation in the promoter region with consequent activation of genes that are transcriptionally repressed under standard conditions (73).

Alterations in DNA methylation are well known in multiple oncological disorders. One of the leading causes of cancer-related mortality worldwide is colorectal cancer (CRC) (74), for which global DNA hypomethylation and depletion of 5-metylcytosine in CRC tissue was reported already in 1983 (75). Such DNA hypomethylation was accompanied by hypermethylation and transcriptional silencing of several tumour suppressor genes (76). Nowadays, it is known that hundreds of genes might be aberrantly methylated in the average CRC genome (77). It is important to keep in mind, that CRC is not a homogenous disease, but rather a heterogeneous disorder with different subtypes characterized by distinct genetic, cytogenetic and epigenetic alterations. Genomic instability is typical for CRC as a key a distinctive characteristic. Genome-scale analysis of aberrant DNA methylation identified four DNA methylation-based subgroups of CRC based on CpG island methylator phenotype (CIMP), specific subgroup of CRC that displays extensive levels of methylated genes (78). The first subgroup is a CIMP-high subgroup exhibiting a very high frequency of cancer-specific DNA hypermethylation, which is associated with MLH1 methylation and the BRAF V100E mutation. The second one is a CIMP-low subgroup associated with K-RAS mutations and characteristic for methylation of a subset of CIMP-high associated genes, rather than a unique group of CpG islands. The third one is a non-CIMP subgroup characterized by TP53 mutations and typical localization in the distal colon. Finally, the last one is a non-CIMP subgroup with low frequency of cancer-specific gene mutations and hypermethylation as well as with rectal localization (79).

To our knowledge, up to date, there has been published no work about the risk of CRC developing in the adulthood, which would be based on delivery mode history. Therefore, further research will be required to answer the question whether or not changes in DNA methylation occurring at the time of delivery could be associated with CRC onset in the adulthood.

There is rising evidence that changes in DNA methylation could be associated not only with physical functions but also with psychosocial functions and neuropsychological disorders. Similar to the above-mentioned hypothesis of stress-induced changes in DNA methylation state upon delivery by CS, several studies have reported that DNA methylation of NR3C1 gene could be affected by epigenetic factors such as quality of maternal care and experience of prenatal and childhood trauma (35, 80–83) which may lead to neuropsychiatric disorders, e.g., depression, anxiety and post-traumatic stress disorder. The mechanism behind these changes is not known but it may be associated with long-lasting dysregulation of the hypothalamus-pituitary-adrenal (84) axis. Hyperactivity of HPA axis induces an increase in secretion of cortisol, a predominant glucocorticoid in humans, which affects the central nervous system by binding to its two receptors encoded by the NR3C1 and NR3C2 genes. These receptors are expressed in the hippocampus, prefrontal cortex and the parvocellular nucleus of the hypothalamus (85). McGowan et al. (86) observed increased DNA methylation of the NR3C1 gene promoter in the hippocampus and prefrontal cortex in suicide victims with a history of childhood abuse. Thus, early-life disruption in the HPA-axis could be mediated by epigenetic mechanisms.

Chronic excess and attenuation of the endogenous diurnal variation in cortisol secretion leads to Cushing’s syndrome (87), which can be caused by an ACTH-producing pituitary adenoma (Cushing’s disease) or by a cortisol-producing adrenal adenoma. In our methylation pathway analysis, both cortisol synthesis and secretion as well as Cushing’s syndrome were identified as pathways with different DNA methylation between VD and CS. Lee et al. (88, 89) showed association of hypomethylation of FKBP5 gene in Cushing’s syndrome patients with anxiety-like behavior. Moreover, such methylation was reduced with glucocorticoid replacement therapy.

In concordance with observed associations between changes in DNA methylome and Cushing’s syndrome, we hypothesize that differences in DNA methylation could play a pathophysiological role in development of stress-related diseases.

Although there are several studies trying to shed more light on the relationship of changes in DNA methylation status and the mode of delivery, the results are inconsistent.

Franz et al. (90) observed hypermethylation of *FOXP3*, *CD7*, *ELA2* and *IRF1* genes for CS and VD samples and significantly higher methylation in *ELA2* and *IRF1* genes in the CS group compared to the VD group. However, global methylation did not differ significantly between the CS and VD groups. Virani et al. (91) observed lower level of global DNA methylation for total CS and planned CS compared to VD but such association disappeared after adjusting for confounding factors. Thus, they concluded that while mode of delivery may be associated with later health outcomes, it does not take effect via changes in global genomic methylation. Similarly, Franz et al. did not report any significant differences in global DNA methylation between CS and VD. However, the global analysis that was used in both papers, reported the average methylation levels in the interrogated restriction sites throughout the whole genome. Therefore, even if the changes were substantial, such method displays only small differences for similar amounts of hyper- and hypomethylation.

On the other hand, Almgren et al. (92), who analyzed CD34^+^ hematopoietic stem cells, found significant effect of mode of delivery on the global DNA methylation pattern. CD34^+^ cells from newborns delivered by CS were globally more DNA-methylated compared to DNA of infants delivered vaginally. They also showed in the locus-specific analysis that the functional relevance of differentially methylated loci involved processes such as immunoglobulin biosynthesis, regulation of glycolysis and ketone metabolism as well as regulation of food response. In another specific gene-targeted analysis, Chen et al. (4) identified 5 genes, whose gene expression level had profound implication for development of the immune system’s response in newborns. Such abnormality of the immune system could lead to chronic inflammation and autoimmune diseases.

Comparison of results across different studies is relatively complicated because of wide range of approaches used for detection of DNA methylation. In comparison with Chen et al., we did not observe any changes in methylation of immune system-related genes and also our methylation pathway analysis did not identify any pathway associated with the immune system. This may be explained by differences between Slovakian and Chinese population. Similarly to Almgren et al., we observed changes in DNA methylation associated with regulation of glucose metabolism, which may have implications for the state of health of the subjects in the future, e.g., because of reported higher risk of obesity and onset of diabetes in subjects with caesarean section in their medical history (93–95). Discrepancies in global DNA methylation between Almgren et al., who observed higher global DNA methylation and Franz et al. as well as Virani et al., who did not report any differences in global DNA methylation, could be explained by specific type of cells (CD34^+^ cells) used in the analysis by Almgren et al. and even here the observed higher level of methylation was relatively small (+2%). Comparison of our results with global DNA methylation could be based on the assumption that this approach delivers the average methylation levels in the interrogated restriction sites throughout the whole genome. Therefore, even if the changes were substantial, such method displays only small differences for similar amounts of hyper- and hypomethylation. Therefore, when Franz et al. evaluated also single gene methylation status, they observed hypermethylation for the group of genes comprising *FOXP3*, *CD7*, *ELA2* and *IRF1*.

Omitting differences in reported DNA methylation at birth that could be affected by the method of evaluation as stated above, it will also be important to clarify whether the observed changes in DNA methylation could be long-lasting with potential of further implications in the future. In this paper, we outlined that some differences in DNA methylation may be amended while others may emerge shortly after delivery. Our results therefore represent a very good opportunity for further research focused on the consequences of differences in DNA methylation on the state of health in adolescence and adult age.

We are aware of some limitations of the present study. One of them is small number of involved subjects and intra-group diversity which could affect representativeness of the target population. Yet another limitation is the negative maternal history of tobacco use, which does not reflect natural distribution. It is also demanding to incorporate the entire range of epigenetic factors with the potential to induce changes in DNA methylation, e.g., medication during gravidity, mother-related health issues as well as complications during labour.

## Conclusion

Our observation of differences in DNA methylation between blood samples from neonates delivered by conventional vaginal delivery and by caesarean section suggests that caesarean section probably represents an important epigenetic factor with the potential to induce changes in the genome. As we showed in our pathway analysis, these differences could play an important role in the development of various type of disorders. Our results could contribute to explaining how epigenetic factors such as mode of delivery could have an adverse impact on the individual’s health later in the life and which mechanisms could be crucial for development of such disorders.

## Data availability statement

The datasets presented in this study can be found in online repositories. The names of the repository/repositories and accession number(s) can be found at: https://www.ebi.ac.uk/ena, PRJEB61787.

## Ethics statement

The studies involving humans were approved by Ethics Committee of the Hospital Agel, Kosice-Saca, Slovakia (EK/2020-06). The studies were conducted in accordance with the local legislation and institutional requirements. Written informed consent for participation in this study was provided by the participants’ legal guardians/next of kin.

## Author contributions

PK: Writing – original draft. DK: Writing – review & editing. DH: Writing – review & editing. OP: Writing – review & editing. KB: Writing – review & editing. ED: Writing – review & editing. ZT: Writing – review & editing. GM: Writing – review & editing.

## Glossary

**Table:** 

A	Adenine
ABAT	4-aminobutyrate aminotransferase
ACACB	Acetyl-CoA carboxylase beta
ACADM	Acyl-CoA dehydrogenase edium chain
ACADVL	Acyl-CoA dehydrogenase very long chain
ACTH	Adrenocorticotropic hormone
ACVR2A	Activin A receptor Type 2A
ADORA2A	Adenosine A2a receptor
ADSS1	Adenylosuccinate synthase 1
AGAP1	ArfGAP with GTPase domain, ankyrin repeat and PH domain 1
AGE	Advanced glycation end product
APOBEC	Apolipoprotein B mRNA editing catalytic polypeptide-like
ARHGEF12	Rho guanine nucleotide exchange factor 12
ASAP2	ArfGAP with SH3 domain, ankyrin repeat and PH domain 2
ATLL	Adult T-cell leukaemia/lymphoma
BOC	BOC cell adhesion associated, oncogene regulated
BCL	Binary base call
BED	Browser extensible data
BMI	Body mass index
bp	Base pair
BRAF	Serine/threonine-protein kinase B-raf
BSSH	BaseSpace Sequence Hub
C	Cytosine
CACNA1H	Calcium voltage-gated channel subunit alpha1 H
CACNA1I	Calcium voltage-gated channel subunit alpha1 I
CACNG7	Calcium voltage-gated channel auxiliary subunit gamma 7
cAMP	Cyclic adenosine 3′,5′monophosphate
CD	Cluster of differentiation
CIMP	CpG island methylator phenotype
CNS	Central nervous system
COL4A2	Collagen type IV alpha 2 chain
CpG	Cytosine phosphate-guanine
CPS1	Carbamoyl-phosphate synthase 1
CRC	Colorectal cancer
CRE	cAMP response elements
CREB	cAMP responsive element binding protein
CREB5	cAMP responsive element binding protein 5
CS	Caesarean section
CTNNA1	Catenin alpha 1
CTNNA2	Catenin alpha 2
CXCR2	C-X-C motif chemokine receptor 2
CYP11B2	Aldosterone synthase
DBCAMP	N6,2′-O-dibutyryladenosine-3′,5′-cyclic monophosphate
DISP1	Dispatched RND transporter family member 1
DNA	Deoxyribonucleic acid
DNMT	DNA methyltransferase
DNM3	Dynamin 3
DOCK1	Dedicator of cytokinesis 1
EGF	Epidermal growth factor
ELA2	Encoding neutrophil elastase
EPN3	Epsin 3
ER-α	Estrogen receptor alpha
EVC	EvC ciliary complex subunit 1
F	Female
FASTQ	Text-based format for storing both a biological sequence and its corresponding quality scores
FKBP5	FK506 binding protein 5
FOXP3	Forkhead box P3
FZD1	Frizzled class receptor 1
FZD1	Frizzled class receptor 1
G	Guanine
GA	Gestational age
GHRHR	Growth hormone releasing hormone receptor
GLI2	GLI family zinc finger 2
GNA11	G protein subunit alpha 11
GNAL	G protein subunit alpha L
GNAS	GNAS complex locus
GNG10	G protein subunit gamma 10
GnRH	Gonadotropin-releasing hormone
GRK5	G protein-coupled receptor kinase 5
GSTM5	Glutathione S-transferase Mu 5
HADH	Hydroxyacyl-CoA dehydrogenase
HADHB	Hydroxyacyl-CoA dehydrogenase trifunctional multienzyme complex subunit beta
HCMV	Human cytomegalovirus
Hg19	Human genome version 19
HIF-1	Hypoxia-inducible factor 1
HK1	Hexokinase 1
HK2	Hexokinase 2
HLA-A	Major histocompatibility complex, class I, A
HLA-G	Major histocompatibility complex, class I, G
HPA	Hypothalamic-pituitary-adrenal
HSPA6	Heat shock protein family A (Hsp70) member 6
HTLV-1	Human T-cell leukaemia virus type 1
CHMP4B	Charged multivesicular body protein 4B
chr	Chromosome
IDO2	Indoleamine 2,3-dioxygenase 2
IGF1R	Insulin like growth factor 1 receptor
IL17RA	Interleukin 17 receptor A
IL17RC	Interleukin 17 receptor C
INMT	Indolethylamine N-methyltransferase
INPP5D	Inositol polyphosphate-5-phosphatase D
IQCE	IQ motif containing E
IQSEC3	IQ motif and Sec7 domain ArfGEF 3
IRF1	Interferon regulatory factor 1
ITPR1	Inositol 1,4,5-trisphosphate receptor type 1
ITPR3	Inositol 1,4,5-trisphosphate receptor type 2
JMJD7-PLA2G4B	JMJD7-PLA2G4B readthrough
KEGG	Kyoto Encyclopaedia of Genes and Genomes
KLC3	Kinesin light chain 3
K-RAS	Kirsten rat sarcoma virus
LAMA2	Laminin subunit alpha 2
LPIN1	Lipin 1
Ly75	Lymphocyte antigen 75
M	Male
MAOA	Monoamine oxidase A
MAP1LC3B2	Microtubule associated protein 1 light chain 3 beta 2
MFN2	Mitofusin 2
MGRN1	Mahogunin ring finger 1
MGST1	Microsomal glutathione S-transferase 1
MLH1	MutL protein homolog 1
MLL3	Mixed-lineage leukaemia protein 3
mRNA	Messenger RNA
ms	Millisecond
MVB12B	Multivesicular body subunit 12B
MYC	MYC proto-oncogene, BHLH transcription factor
Na/K-ATP-ase	Sodium-potassium-ATPase
NDUFA9	NADH: ubiquinone oxidoreductase subunit A9
NEDD4L	NEDD4 Llke E3 ubiquitin protein ligase
NFATC1	Nuclear factor of activated T cells 1
NFKBIB	NFKB inhibitor beta
NGS	Next-generating sequencing
NOS3	Nitric oxide synthase 3
NR3C1	Nuclear receptor subfamily 3, group C, member 1
NR3C2	Nuclear receptor subfamily 3, group C, member 2
PCR	Polymerase chain reaction
PDGF	Platelet derived growth factor
PDGFRA	Platelet derived growth factor receptor alpha
PDGR	Platelet-derived growth factor
PFKP	Phosphofructokinase, platelet
PLA2G4B	Phospholipase A2 group IVB
PLCE1	Phospholipase C epsilon 1
PLCG2	Phospholipase C gamma 2
PLD2	Phospholipase D2
POLK	DNA polymerase kappa
PRKCA	Protein kinase C alpha
PRKCB	Protein kinase C beta
PRKCE	Protein kinase C epsilon
PSD4	Pleckstrin and Sec7 domain containing 4
PSMD1	Proteasome 26S subunit, non-ATPase 1
PTEN	Phosphatase and tensin homolog
qPCR	Quantitative polymerase chain reaction
RAB31	RAB31, member RAS oncogene family
RAB5A	RAB5A, member RAS oncogene family
RAGE	Advanced glycation end product (AGE) receptor
RNA	Ribonucleic acid
SCIN	Scinderin
SD	Standard deviation
SDHA	Succinate dehydrogenase complex flavoprotein subunit A
SERPINA5	Serpin family a member 5
SGCA	Sarcoglycan alpha
SLC18A2	Solute carrier family 18 member A2
SMAD3	SMAD family member 3
SPHK1	Sphingosine kinase 1
SSTR5	Somatostatin receptor 5
STAM	Signal transducing adaptor molecule
SYK	Spleen associated tyrosine kinase
T	Thymine
TBXAS1	Thromboxane A synthase 1
TCF7L1	Transcription factor 7 like 1
TET	Tet methylcytosine dioxygenase
TFDP1	Transcription factor Dp-1
TGF	Transforming growth factor
TGFBR2	Transforming growth factor beta receptor 2
TGIF1	TGFB induced factor homeobox 1
TGIF2	TGFB induced factor homeobox 2
THSD4	Thrombospondin type 1 domain containing 4
TP53	Tumour protein p53
TP73	Tumour protein P73
TPM4	Tropomyosin 4
TRPM2	Transient receptor potential cation channel subfamily M member 2
VD	Vaginal delivery
VEGFA	Vascular endothelial growth factor A
VSMC	Vascular smooth muscle cell
WNT8A	Wnt family member 8A
WWC1	WW and C2 domain containing 1

## References

[1] Betrán AP Ye J Moller AB Zhang J Gülmezoglu AM Torloni MR. The increasing trend in caesarean section rates: global, regional and national estimates: 1990–2014. PLoS One. (2016) 11:e0148343. doi: 10.1371/journal.pone.0148343, PMID: 26849801 PMC4743929

[2] Betran AP Ye J Moller AB Souza JP Zhang J. Trends and projections of caesarean section rates: global and regional estimates. BMJ Glob Health. (2021) 6:e005671. doi: 10.1136/bmjgh-2021-005671, PMID: 34130991 PMC8208001

[3] Betran AP Torloni MR Zhang JJ Gülmezoglu AM The WHO Working Group on Caesarean Section. WHO statement on caesarean section rates. BJOG. (2016) 123:667–70. doi: 10.1111/1471-0528.13526, PMID: 26681211 PMC5034743

[4] Chen Q Ming Y Gan Y Huang L Zhao Y Wang X. The impact of cesarean delivery on infant DNA methylation. BMC Pregnancy Childbirth. (2021) 21:265. doi: 10.1186/s12884-021-03748-y, PMID: 33785011 PMC8011183

[5] Lupu VV Miron IC Raileanu AA Starcea IM Lupu A Tarca E. Difficulties in adaptation of the mother and newborn via cesarean section versus natural birth-a narrative review. Life. (2023) 13:300. doi: 10.3390/life13020300, PMID: 36836657 PMC9965845

[6] Chiș A Vulturar R Andreica S Prodan A Miu AC. Behavioral and cortisol responses to stress in newborn infants: effects of mode of delivery. Psychoneuroendocrinology. (2017) 86:203–8. doi: 10.1016/j.psyneuen.2017.09.024, PMID: 28987898

[7] Chen DC Nommsen-Rivers L Dewey KG Lönnerdal B. Stress during labor and delivery and early lactation performance. Am J Clin Nutr. (1998) 68:335–44. doi: 10.1093/ajcn/68.2.335, PMID: 9701191

[8] Miller NM Fisk NM Modi N Glover V. Stress responses at birth: determinants of cord arterial cortisol and links with cortisol response in infancy. BJOG. (2005) 112:921–6. doi: 10.1111/j.1471-0528.2005.00620.x, PMID: 15957993

[9] Malamitsi-Puchner A Protonotariou E Boutsikou T Makrakis E Sarandakou A Creatsas G. The influence of the mode of delivery on circulating cytokine concentrations in the perinatal period. Early Hum Dev. (2005) 81:387–92. doi: 10.1016/j.earlhumdev.2004.10.017, PMID: 15814224

[10] Heasman L Spencer JA Symonds ME. Plasma prolactin concentrations after caesarean section or vaginal delivery. Arch Dis Child Fetal Neonatal Ed. (1997) 77:F237–8. doi: 10.1136/fn.77.3.F237, PMID: 9462197 PMC1720725

[11] Lubetzky R Ben-Shachar S Mimouni F Dollberg S. Mode of delivery and neonatal hematocrit. Am J Perinatol. (2000) 17:163–6. doi: 10.1055/s-2000-9291 11012142

[12] Biasucci G Rubini M Riboni S Morelli L Bessi E Retetangos C. Mode of delivery affects the bacterial community in the newborn gut. Early Hum Dev. (2010) 86:13–5. doi: 10.1016/j.earlhumdev.2010.01.004, PMID: 20133091

[13] Yektaei-Karin E Moshfegh A Lundahl J Berggren V Hansson LO Marchini G. The stress of birth enhances in vitro spontaneous and IL-8-induced neutrophil chemotaxis in the human newborn. Pediatr Allergy Immunol. (2007) 18:643–51. doi: 10.1111/j.1399-3038.2007.00578.x, PMID: 18078418

[14] Curran EA Dalman C Kearney PM Kenny LC Cryan JF Dinan TG. Association between obstetric mode of delivery and autism spectrum disorder: a population-based sibling design study. JAMA Psychiatry. (2015) 72:935–42. doi: 10.1001/jamapsychiatry.2015.0846, PMID: 26107922

[15] O’Neill SM Curran EA Dalman C Kenny LC Kearney PM Clarke G. Birth by caesarean section and the risk of adult psychosis: a population-based cohort study. Schizophr Bull. (2016) 42:633–41. doi: 10.1093/schbul/sbv152, PMID: 26615187 PMC4838084

[16] Hyde MJ Modi N. The long-term effects of birth by caesarean section: the case for a randomised controlled trial. Early Hum Dev. (2012) 88:943–9. doi: 10.1016/j.earlhumdev.2012.09.006, PMID: 23036493

[17] Thavagnanam S Fleming J Bromley A Shields MD Cardwell CR. A meta-analysis of the association between caesarean section and childhood asthma. Clin Exp Allergy. (2008) 38:629–33. doi: 10.1111/j.1365-2222.2007.02780.x, PMID: 18352976

[18] Dahlen HG Downe S Wright ML Kennedy HP Taylor JY. Childbirth and consequent atopic disease: emerging evidence on epigenetic effects based on the hygiene and EPIIC hypotheses. BMC Pregnancy Childbirth. (2016) 16:4. doi: 10.1186/s12884-015-0768-9, PMID: 26762406 PMC4712556

[19] Decker E Engelmann G Findeisen A Gerner P Laaβ M Ney D. Cesarean delivery is associated with celiac disease but not inflammatory bowel disease in children. Pediatrics. (2010) 125:e1433–40. doi: 10.1542/peds.2009-2260, PMID: 20478942

[20] Cardwell CR Stene LC Joner G Cinek O Svensson J Goldacre MJ. Caesarean section is associated with an increased risk of childhood-onset type 1 diabetes mellitus: a meta-analysis of observational studies. Diabetologia. (2008) 51:726–35. doi: 10.1007/s00125-008-0941-z, PMID: 18292986

[21] Sevelsted A Stokholm J Bønnelykke K Bisgaard H. Cesarean section and chronic immune disorders. Pediatrics. (2015) 135:e92–8. doi: 10.1542/peds.2014-0596 25452656

[22] Keag OE Norman JE Stock SJ. Long-term risks and benefits associated with cesarean delivery for mother, baby, and subsequent pregnancies: systematic review and meta-analysis. PLoS Med. (2018) 15:e1002494. doi: 10.1371/journal.pmed.1002494, PMID: 29360829 PMC5779640

[23] Sandoval J Heyn H Moran S Serra-Musach J Pujana MA Bibikova M. Validation of a DNA methylation microarray for 450,000 CpG sites in the human genome. Epigenetics. (2011) 6:692–702. doi: 10.4161/epi.6.6.16196 21593595

[24] Rechache NS Wang Y Stevenson HS Killian JK Edelman DC Merino M. DNA methylation profiling identifies global methylation differences and markers of adrenocortical tumors. J Clin Endocrinol Metab. (2012) 97:E1004–13. doi: 10.1210/jc.2011-3298, PMID: 22472567 PMC3387424

[25] Jirtle RL Skinner MK. Environmental epigenomics and disease susceptibility. Nat Rev Genet. (2007) 8:253–62. doi: 10.1038/nrg2045, PMID: 17363974 PMC5940010

[26] Smallwood SA Kelsey G. *De novo* DNA methylation: a germ cell perspective. Trends Genet. (2012) 28:33–42. doi: 10.1016/j.tig.2011.09.004, PMID: 22019337

[27] Cedar H Bergman Y. Programming of DNA methylation patterns. Annu Rev Biochem. (2012) 81:97–117. doi: 10.1146/annurev-biochem-052610-091920 22404632

[28] Reizel Y Sabag O Skversky Y Spiro A Steinberg B Bernstein D. Postnatal DNA demethylation and its role in tissue maturation. Nat Commun. (2018) 9:2040. doi: 10.1038/s41467-018-04456-6, PMID: 29795194 PMC5966414

[29] Ziller MJ Gu H Müller F Donaghey J Tsai LTY Kohlbacher O. Charting a dynamic DNA methylation landscape of the human genome. Nature. (2013) 500:477–81. doi: 10.1038/nature12433, PMID: 23925113 PMC3821869

[30] Hon GC Rajagopal N Shen Y McCleary DF Yue F Dang MD. Epigenetic memory at embryonic enhancers identified in DNA methylation maps from adult mouse tissues. Nat Genet. (2013) 45:1198–206. doi: 10.1038/ng.2746, PMID: 23995138 PMC4095776

[31] Sheaffer KL Kim R Aoki R Elliott EN Schug J Burger L. DNA methylation is required for the control of stem cell differentiation in the small intestine. Genes Dev. (2014) 28:652–64. doi: 10.1101/gad.230318.113, PMID: 24637118 PMC3967052

[32] Cannon MV Pilarowski G Liu X Serre D. Extensive epigenetic changes accompany terminal differentiation of mouse hepatocytes after birth. G3. (2016) 6:3701–9. doi: 10.1534/g3.116.034785, PMID: 27652892 PMC5100869

[33] Ehara T Kamei Y Yuan X Takahashi M Kanai S Tamura E. Ligand-activated PPARα-dependent DNA demethylation regulates the fatty acid beta-oxidation genes in the postnatal liver. Diabetes. (2015) 64:775–84. doi: 10.2337/db14-0158, PMID: 25311726

[34] Reizel Y Spiro A Sabag O Skversky Y Hecht M Keshet I. Gender-specific postnatal demethylation and establishment of epigenetic memory. Genes Dev. (2015) 29:923–33. doi: 10.1101/gad.259309.115, PMID: 25934504 PMC4421981

[35] Weaver IC Cervoni N Champagne FA D’Alessio AC Sharma S Seckl JR. Epigenetic programming by maternal behavior. Nat Neurosci. (2004) 7:847–54. doi: 10.1038/nn1276 15220929

[36] Ganesh S Cave V. P-values, p-values everywhere!. N Z Vet J,. (2018) 66:55–56. 29228893 10.1080/00480169.2018.1415604

[37] Reimand J Kull M Peterson H Hansen J Vilo J. G:profiler—a web-based toolset for functional profiling of gene lists from large-scale experiments. Nucleic Acids Res. (2007) 35:W193–200. doi: 10.1093/nar/gkm226, PMID: 17478515 PMC1933153

[38] Barton M Forrester AM McDonald J. Update on congenital cytomegalovirus infection: prenatal prevention, newborn diagnosis, and management. Paediatr Child Health. (2020) 25:395–6. doi: 10.1093/pch/pxaa083, PMID: 32968468 PMC7492632

[39] Buxmann H Hamprecht K Meyer-Wittkopf M Friese K. Primary human cytomegalovirus (HCMV) infection in pregnancy. Dtsch Arztebl Int. (2017) 114:45–52. doi: 10.3238/arztebl.2017.0045, PMID: 28211317 PMC5319378

[40] Esteki-Zadeh A Karimi M Strååt K Ammerpohl O Zeitelhofer M Jagodic M. Human cytomegalovirus infection is sensitive to the host cell DNA methylation state and alters global DNA methylation capacity. Epigenetics. (2012) 7:585–93. doi: 10.4161/epi.20075, PMID: 22595877

[41] Cardoso MC Leonhardt H. DNA methyltransferase is actively retained in the cytoplasm during early development. J Cell Biol. (1999) 147:25–32. doi: 10.1083/jcb.147.1.25, PMID: 10508852 PMC2164986

[42] Verma M. Viral genes and methylation. Ann N Y Acad Sci. (2003) 983:170–80. doi: 10.1111/j.1749-6632.2003.tb05972.x 12724222

[43] Gessain A Mahieux R. Tropical spastic paraparesis and HTLV-1 associated myelopathy: clinical, epidemiological, virological and therapeutic aspects. Rev Neurol. (2012) 168:257–69. doi: 10.1016/j.neurol.2011.12.006, PMID: 22405461

[44] Kataoka K Shiraishi Y Takeda Y Sakata S Matsumoto M Nagano S. Aberrant PD-L1 expression through 3′-UTR disruption in multiple cancers. Nature. (2016) 534:402–6. doi: 10.1038/nature18294, PMID: 27281199

[45] Yeh CH Bai XT Moles R Ratner L Waldmann TA Watanabe T. Mutation of epigenetic regulators TET2 and MLL3 in patients with HTLV-I-induced acute adult T-cell leukemia. Mol Cancer. (2016) 15:15. doi: 10.1186/s12943-016-0500-z, PMID: 26880370 PMC4754821

[46] Rosadas C Taylor GP. Mother-to-child HTLV-1 transmission: unmet research needs. Front Microbiol. (2019) 10:999. doi: 10.3389/fmicb.2019.00999, PMID: 31134031 PMC6517543

[47] Ying AK Hassanain HH Roos CM Smiraglia DJ Issa JJ Michler RE. Methylation of the estrogen receptor-alpha gene promoter is selectively increased in proliferating human aortic smooth muscle cells. Cardiovasc Res. (2000) 46:172–9. doi: 10.1016/S0008-6363(00)00004-3, PMID: 10727665

[48] Sharma P Garg G Kumar A Mohammad F Kumar SR Tanwar VS. Genome wide DNA methylation profiling for epigenetic alteration in coronary artery disease patients. Gene. (2014) 541:31–40. doi: 10.1016/j.gene.2014.02.034, PMID: 24582973

[49] Sharma P Kumar J Garg G Kumar A Patowary A Karthikeyan G. Detection of altered global DNA methylation in coronary artery disease patients. DNA Cell Biol. (2008) 27:357–65. doi: 10.1089/dna.2007.0694 18613790

[50] Haas J Frese KS Park YJ Keller A Vogel B Lindroth AM. Alterations in cardiac DNA methylation in human dilated cardiomyopathy. EMBO Mol Med. (2013) 5:413–29. doi: 10.1002/emmm.201201553, PMID: 23341106 PMC3598081

[51] Koczor CA Lee EK Torres RA Boyd A Vega JD Uppal K. Detection of differentially methylated gene promoters in failing and nonfailing human left ventricle myocardium using computation analysis. Physiol Genomics. (2013) 45:597–605. doi: 10.1152/physiolgenomics.00013.2013, PMID: 23695888 PMC3727018

[52] Movassagh M Choy MK Goddard M Bennett MR Down TA Foo RSY. Differential DNA methylation correlates with differential expression of angiogenic factors in human heart failure. PLoS One. (2010) 5:e8564. doi: 10.1371/journal.pone.0008564, PMID: 20084101 PMC2797324

[53] Movassagh M Choy MK Knowles DA Cordeddu L Haider S Down T. Distinct epigenomic features in end-stage failing human hearts. Circulation. (2011) 124:2411–22. doi: 10.1161/CIRCULATIONAHA.111.040071, PMID: 22025602 PMC3634158

[54] Frismantiene A Philippova M Erne P Resink TJ. Smooth muscle cell-driven vascular diseases and molecular mechanisms of VSMC plasticity. Cell Signal. (2018) 52:48–64. doi: 10.1016/j.cellsig.2018.08.019, PMID: 30172025

[55] Chi C Li DJ Jiang YJ Tong J Fu H Wu YH. Vascular smooth muscle cell senescence and age-related diseases: state of the art. Biochim Biophys Acta Mol basis Dis. (2019) 1865:1810–21. doi: 10.1016/j.bbadis.2018.08.015, PMID: 31109451

[56] Lacolley P Regnault V Avolio AP. Smooth muscle cell and arterial aging: basic and clinical aspects. Cardiovasc Res. (2018) 114:513–28. doi: 10.1093/cvr/cvy009, PMID: 29514201

[57] Zhang D Chen Y Xie X Liu J Wang Q Kong W. Homocysteine activates vascular smooth muscle cells by DNA demethylation of platelet-derived growth factor in endothelial cells. J Mol Cell Cardiol. (2012) 53:487–96. doi: 10.1016/j.yjmcc.2012.07.010, PMID: 22867875

[58] Wu L Pei Y Zhu Y Jiang M Wang C Cui W. Association of N^6^-methyladenine DNA with plaque progression in atherosclerosis via myocardial infarction-associated transcripts. Cell Death Dis. (2019) 10:909. doi: 10.1038/s41419-019-2152-6, PMID: 31797919 PMC6892866

[59] Xu L Hao H Hao Y Wei G Li G Ma P. Aberrant MFN2 transcription facilitates homocysteine-induced VSMCs proliferation via the increased binding of c-Myc to DNMT1 in atherosclerosis. J Cell Mol Med. (2019) 23:4611–26. doi: 10.1111/jcmm.14341, PMID: 31104361 PMC6584594

[60] Ma SC Zhang HP Jiao Y Wang YH Zhang H Yang XL. Homocysteine-induced proliferation of vascular smooth muscle cells occurs via PTEN hypermethylation and is mitigated by resveratrol. Mol Med Rep. (2018) 17:5312–9. doi: 10.3892/mmr.2018.8471, PMID: 29393420

[61] Min J Weitian Z Peng C Yan P Bo Z Yan W. Correlation between insulin-induced estrogen receptor methylation and atherosclerosis. Cardiovasc Diabetol. (2016) 15:156. doi: 10.1186/s12933-016-0471-9, PMID: 27832775 PMC5105242

[62] Liu R Jin Y Tang WH Qin L Zhang X Tellides G. Ten-eleven translocation-2 (TET2) is a master regulator of smooth muscle cell plasticity. Circulation. (2013) 128:2047–57. doi: 10.1161/CIRCULATIONAHA.113.002887, PMID: 24077167 PMC3899790

[63] Hiltunen MO Turunen MP Häkkinen TP Rutanen J Hedman M Mäkinen K. DNA hypomethylation and methyltransferase expression in atherosclerotic lesions. Vasc Med. (2002) 7:5–11. doi: 10.1191/1358863x02vm418oa, PMID: 12083735

[64] Pepin ME Drakos S Ha CM Tristani-Firouzi M Selzman CH Fang JC. DNA methylation reprograms cardiac metabolic gene expression in end-stage human heart failure. Am J Physiol Heart Circ Physiol. (2019) 317:H674–84. doi: 10.1152/ajpheart.00016.2019, PMID: 31298559 PMC6843013

[65] Arai K Papadopoulou-Marketou N Chrousos GP. Aldosterone deficiency and resistance In: Feingold KR Anawalt B Blackman MR, editors. Endotext. South Dartmouth, MA: Mdtext Com Inc. (2000) 25905305

[66] White PC. Disorders of aldosterone biosynthesis and action. N Engl J Med. (1994) 331:250–8. doi: 10.1056/NEJM199407283310408, PMID: 8015573

[67] Demura M Bulun SE. CpG dinucleotide methylation of the CYP19 I.3/II promoter modulates cAMP-stimulated aromatase activity. Mol Cell Endocrinol. (2008) 283:127–32. doi: 10.1016/j.mce.2007.12.003, PMID: 18201819

[68] Takeda Y Demura M Wang F Karashima S Yoneda T Kometani M. Epigenetic regulation of aldosterone synthase gene by sodium and angiotensin II. J Am Heart Assoc. (2018) 7:e008281. doi: 10.1161/JAHA.117.008281 29739797 PMC6015301

[69] Zhang P Li T Liu YQ Zhang H Xue SM Li G. Contribution of DNA methylation in chronic stress-induced cardiac remodeling and arrhythmias in mice. FASEB J. (2019) 33:12240–52. doi: 10.1096/fj.201900100R, PMID: 31431066 PMC6902688

[70] Xiao J Sluijter JP Das S Yang Y Shen Z. A snapshot of genetic and epigenetic basis of arrhythmia and heart failure. Front Genet. (2015) 6:74. doi: 10.3389/fgene.2015.00074 25784925 PMC4347482

[71] Daniel PB Walker WH Habener JF. Cyclic AMP signaling and gene regulation. Annu Rev Nutr. (1998) 18:353–83. doi: 10.1146/annurev.nutr.18.1.353 9706229

[72] Shaywitz AJ Greenberg ME. CREB: a stimulus-induced transcription factor activated by a diverse array of extracellular signals. Annu Rev Biochem. (1999) 68:821–61. doi: 10.1146/annurev.biochem.68.1.821, PMID: 10872467

[73] Kass SU Pruss D Wolffe AP. How does DNA methylation repress transcription? Trends Genet. (1997) 13:444–9. doi: 10.1016/S0168-9525(97)01268-7 9385841

[74] Sung H Ferlay J Siegel RL Laversanne M Soerjomataram I Jemal A. Global cancer statistics 2020: GLOBOCAN estimates of incidence and mortality worldwide for 36 cancers in 185 countries. CA Cancer J Clin. (2021) 71:209–49. doi: 10.3322/caac.21660, PMID: 33538338

[75] Feinberg AP Vogelstein B. Hypomethylation distinguishes genes of some human cancers from their normal counterparts. Nature. (1983) 301:89–92. doi: 10.1038/301089a0, PMID: 6185846

[76] Feinberg AP Vogelstein B. Hypomethylation of ras oncogenes in primary human cancers. Biochem Biophys Res Commun. (1983) 111:47–54. doi: 10.1016/S0006-291X(83)80115-6, PMID: 6187346

[77] Lao VV Grady WM. Epigenetics and colorectal cancer. Nat Rev Gastroenterol Hepatol. (2011) 8:686–700. doi: 10.1038/nrgastro.2011.173, PMID: 22009203 PMC3391545

[78] Toyota M Ahuja N Ohe-Toyota M Herman JG Baylin SB Issa JPJ. CpG island methylator phenotype in colorectal cancer. Proc Natl Acad Sci U S A. (1999) 96:8681–6. doi: 10.1073/pnas.96.15.8681, PMID: 10411935 PMC17576

[79] Hinoue T Weisenberger DJ Lange CPE Shen H Byun HM van den Berg D. Genome-scale analysis of aberrant DNA methylation in colorectal cancer. Genome Res. (2012) 22:271–82. doi: 10.1101/gr.117523.110, PMID: 21659424 PMC3266034

[80] Hompes T Izzi B Gellens E Morreels M Fieuws S Pexsters A. Investigating the influence of maternal cortisol and emotional state during pregnancy on the DNA methylation status of the glucocorticoid receptor gene (NR3C1) promoter region in cord blood. J Psychiatr Res. (2013) 47:880–91. doi: 10.1016/j.jpsychires.2013.03.009, PMID: 23566423

[81] Mulligan CJ D’Errico N Stees J Hughes D. Methylation changes at NR3C1 in newborns associate with maternal prenatal stress exposure and newborn birth weight. Epigenetics. (2012) 7:853–7. doi: 10.4161/epi.21180, PMID: 22810058 PMC3427280

[82] Oberlander TF Weinberg J Papsdorf M Grunau R Misri S Devlin AM. Prenatal exposure to maternal depression, neonatal methylation of human glucocorticoid receptor gene (NR3C1) and infant cortisol stress responses. Epigenetics. (2008) 3:97–106. doi: 10.4161/epi.3.2.6034, PMID: 18536531

[83] Romens SE McDonald J Svaren J Pollak SD. Associations between early life stress and gene methylation in children. Child Dev. (2015) 86:303–9. doi: 10.1111/cdev.12270, PMID: 25056599 PMC4305348

[84] Mahajan HB Rashid AS Junnarkar AA Uke N Deshpande SD Futane PR. Integration of Healthcare 4.0 and blockchain into secure cloud-based electronic health records systems. Appl Nanosci. (2022) 13:2329–42. doi: 10.1007/s13204-021-02164-0 35136707 PMC8813573

[85] Reul JM de Kloet ER. Two receptor systems for corticosterone in rat brain: microdistribution and differential occupation. Endocrinology. (1985) 117:2505–11. doi: 10.1210/endo-117-6-2505, PMID: 2998738

[86] McGowan PO Sasaki A D’Alessio AC Dymov S Labonté B Szyf M. Epigenetic regulation of the glucocorticoid receptor in human brain associates with childhood abuse. Nat Neurosci. (2009) 12:342–8. doi: 10.1038/nn.2270, PMID: 19234457 PMC2944040

[87] Newell-Price J Bertagna X Grossman AB Nieman LK. Cushing’s syndrome. Lancet. (2006) 367:1605–17. doi: 10.1016/S0140-6736(06)68699-6 16698415

[88] Lee RS Tamashiro KLK Yang X Purcell RH Harvey A Willour VL. Chronic corticosterone exposure increases expression and decreases deoxyribonucleic acid methylation of Fkbp5 in mice. Endocrinology. (2010) 151:4332–43. doi: 10.1210/en.2010-0225, PMID: 20668026 PMC2940504

[89] Lee RS Tamashiro KLK Yang X Purcell RH Huo Y Rongione M. A measure of glucocorticoid load provided by DNA methylation of Fkbp5 in mice. Psychopharmacology. (2011) 218:303–12. doi: 10.1007/s00213-011-2307-3, PMID: 21509501 PMC3918452

[90] Franz MB Poterauer M Elhenicky M Stary S Birner P Vinatzer U. Global and single gene DNA methylation in umbilical cord blood cells after elective caesarean: a pilot study. Eur J Obstet Gynecol Reprod Biol. (2014) 179:121–4. doi: 10.1016/j.ejogrb.2014.05.038, PMID: 24960239

[91] Virani S Dolinoy DC Halubai S Jones TR Domino SE Rozek LS. Delivery type not associated with global methylation at birth. Clin Epigenetics. (2012) 4:8. doi: 10.1186/1868-7083-4-8, PMID: 22682523 PMC3404951

[92] Almgren M Schlinzig T Gomez-Cabrero D Gunnar A Sundin M Johansson S. Cesarean delivery and hematopoietic stem cell epigenetics in the newborn infant: implications for future health? Am J Obstet Gynecol. (2014) 211:502.e1. doi: 10.1016/j.ajog.2014.05.014 24996659

[93] Jensen ET Bertoni AG Crago OL Rotter JI Chen YDI Wood A. Cesarean delivery and insulin sensitivity in the older adult: the microbiome and insulin longitudinal evaluation study. J Endocr Soc. (2022) 6:bvac072. doi: 10.1210/jendso/bvac072 35673403 PMC9165426

[94] Chavarro JE Martín-Calvo N Yuan C Arvizu M Rich-Edwards JW Michels KB. Association of birth by cesarean delivery with obesity and type 2 diabetes among adult women. JAMA Netw Open. (2020) 3:e202605. doi: 10.1001/jamanetworkopen.2020.2605, PMID: 32282045 PMC7154804

[95] Dal’Maso E Rodrigues PRM Ferreira MG Moreira NF Muraro AP. Cesarean birth and risk of obesity from birth to adolescence: a cohort study. Birth. (2022) 49:774–82. doi: 10.1111/birt.12644, PMID: 35527364

